# Adding Chinese herbal medicine to conventional therapy brings cognitive benefits to patients with Alzheimer’s disease: a retrospective analysis

**DOI:** 10.1186/s12906-017-2040-5

**Published:** 2017-12-13

**Authors:** Jing Shi, Jingnian Ni, Tao Lu, Xuekai Zhang, Mingqing Wei, Ting Li, Weiwei Liu, Yongyan Wang, Yuanyuan Shi, Jinzhou Tian

**Affiliations:** 1 BUCM Neurology Centre at Dongzhimen Hospital, Beijing University of Chinese Medicine, No. 5 Haiyuncang Street, Dongcheng, Bejing, 100700 China; 2 School of Life Sciences, Beijing University of Chinese Medicine, No. 11 East road, North 3rd Ring Road, Beijing, 100029 China; 3 Consulting Center of Biomedical Statistics, The Academy of Military Medical Sciences, Beijing, 100850 China; 4 Institute of Basic Research in Clinical Medicine, China Academy of Chinese Medical Sciences, Beijing, 100700 China

**Keywords:** Alzheimer’s disease, Complementary and alternative medicine, Traditional Chinese medicine

## Abstract

**Background:**

Conventional therapy (CT) such as donepezil and memantine are well-known short-term treatments for the symptoms of Alzheimer’s disease (AD). The efficacy of them, however, drops below baseline level after 9 months. In China, herbal therapy as a complementary therapy is very popular. Should conventional therapy combined with herbal therapy (CT + H) make add-on benefit?

**Methods:**

In this retrospective cohort study, 344 outpatients diagnosed as probable dementia due to AD were collected, with the treatment of either CT + H or CT alone in clinical settings. All the patients were examined with coronary MRI scan. Cognitive functions were obtained by mini–mental state examination (MMSE) every 3 months with the longest follow-up of 24 months.

**Results:**

Most of the patients were initially diagnosed with mild (MMSE = 21–26, *n* = 177) and moderate (MMSE = 10–20, *n* = 137) dementia. At 18 months, CT+ H patients scored on average 1.76 (*P* = 0.002) better than CT patients, and at 24 months, patients scored on average 2.52 (*P* < 0.001) better. At 24 months, the patients with improved cognitive function (△MMSE ≥ 0) in CT + H was more than CT alone (33.33% vs 7.69%, *P* = 0.020). Interestingly, patients with mild AD received the most robust benefit from CT + H therapy. The deterioration of the cognitive function was largely prevented at 24 months (ΔMMSE = −0.06), a significant improvement from CT alone (ΔMMSE = −2.66, *P* = 0.005).

**Conclusions:**

Compared to CT alone, CT + H significantly benefited AD patients. A symptomatic effect of CT + H was more pronounced with time. Cognitive decline was substantially decelerated in patients with moderate severity, while the cognitive function was largely stabilized in patients with mild severity over two years. These results imply that Chinese herbal medicines may provide an alternative and additive treatment for AD.

## Background

Alzheimer’s disease (AD) is a growing challenge for global health and economy. AD now affects about 6.25/1000 people per year in China [1]. Approved pharmacotherapies for AD include three cholinesterase inhibitors (ChEIs, such as donepezil, rivastigmine and galantamine) and a N-methyl D-aspartate (NMDA) receptor antagonist (memantine). These well-studied conventional treatments for AD are generally considered to be symptom-relieving rather than disease-modifying. Studies have shown that ChEI treatment for mild to moderate AD may reach peak benefit for cognitive improvement within 3 months, but drop below baseline level after 9 months [2, 3]. Patients with moderate to severe AD receiving stable doses of both donepezil and memantine experienced limited cognitive improvement [4]. In another study, no significant benefit was found by combining donepezil and memantine treatment [5].

On the other hand, herbal medicine has long been used for the treatment of dementia in China [6]. Its effectiveness has not yet been well documented. Previous results from a small short-term clinical trial demonstrated that Chinese herbal medicine has potent cognitive enhancing effects [7]. In our study, over the span of two years, we investigated the effectiveness of combining conventional therapy of ChEI or NMDA antagonist with Chinese herbal medicine on ameliorating cognitive functions in AD patients.

## Methods

### Study design and data collection

It’s a retrospective cohort study and patients diagnosed as Alzheimer’s dementia were collected [8, 9]. Medical records between May 2011 to August 2016 were accessed by using administrative datasets of memory clinic. Clinical records covered detailed medical history, cognitive and neuropsychological tests, neurological examinations, results of laboratory tests (i.e. thyroid function, folic acid levels, vitamin B12 levels, and routine blood tests), and neuroimaging. Hippocampus atrophy was accessed by neuroimaging specialist according to age-adjusted medial temporal lobe atrophy scale (MTA-scale) based on coronary MRI scan of the brain.

The operational definition of Alzheimer’s dementia was as follows: (1) gradual and progressive change in cognitive functions over more than 6 months; (2) objective evidence of significant impairment in episodic memory together with at least one of other cognitive domains; [10] (3) global cognitive decline measured by mini-mental state examination (MMSE) adjusted for education: ≤22 for illiteracy, ≤23 for primary school, ≤24 for middle school,≤26 for high education; [11] (4) impaired abilities of daily living, ADL scale score ≥ 16; [12] (5) ≤4 point on Hachinski Ischaemic Score (HIS); (6) age-adjusted MTA-scale based on coronary magnetic resonance imaging (MRI) scan of the brain (1.0 or more for ≤65 years; 1.5 or more for ≤75 years and 2.0 or more for ≥75 years); [9, 13] (7) other causes of dementia excluded.

Patients with the following characteristics were excluded: (1) sudden onset of cognitive disorder with focal nervous system signs in the early stages of disease, (e.g., incomplete paralysis, anesthesia, dysfunctional visual field, and dystaxia); (2) early occurrence of the following symptoms: gait disturbances, seizures, extrapyramidal signs, hallucinations and cognitive fluctuations; (3) any major psychiatric disorders (e.g., DSM-IV-defined psychosis, major depression, bipolar disorder, or alcohol or substance abuse); (4) other conditions that may explain cognitive impairment (e.g., hypothyroidism, electrolyte imbalance, toxic, inflammatory, and metabolic disorders).

### Interventions and outcome measurements

According to the received treatments, patients were grouped into conventional therapy with herbal medicine (CT + H) or conventional therapy without herbal medicine (CT). Conventional therapy included treatment with Donepezil and/or memantine. Donepezil was used to treat mild to severe AD patients and memantine was given to moderate and severe AD patients. The dose of donepezil ranged from 5 to 10 mg once a day according to patients’ tolerance. Most patients received 10 mg memantine once a day, those with apparent mental and behavior symptoms received up to 20 mg of memantine once a day.

Herbal medicine as a traditional therapy is believed to be helpful for dementia in China. The herbal granule was approved by CFDA for clinical use. The GRAPE formula was prescribed for AD patients after every visit according to TCM theory. It consisted mainly of Ren shen (*Panax* ***g*** *inseng*, 10 g/d), Di huang (**R**ehmannia glutinosa, 30 g/d), Cang pu (**A**corus tatarinowii, 10 g/d), Yuan zhi (**P**olygala tenuifolia, 10 g/d), Yin yanghuo (**E**pimedium brevicornu, 10 g/d), Shan zhuyu (*Cornus officinalis*, 10 g/d), Rou congrong (*Cistanche deserticola*, 10 g/d), Yu jin (*Curcuma aromatica*, 10 g/d), Dan shen (Salvia miltiorrhiza, 10 g/d), Dang gui (Angelica sinensis, 10 g/d), Tian ma (Gastrodia elata, 10 g/d), and Huang lian (Coptis chinensis, 10 g/d), supplied by Beijing Tcmages Pharmaceutical Co., LTD. Daily dose was taken twice and dissolved in 150 ml hot water each time.

Global cognitive function was evaluated mainly with MMSE at 3 (±1.5) month interval, conducted by physicians special in psychological assessment. The data followed-up 24 months was involved in this study. In the mini–mental state examination, the range of scores was 0 to 30, and higher scores indicated better cognitive function.

### Statistical analysis

The statistical analyses were conducted using SAS 9.2. Average continuous variables with standard deviation were calculated when data are normally distributed, and the counting categorical variables were shown in percentage. The demographics between two groups were compared. ANOVA was used for testing the differences of continuous variables when data are normally distributed and equal variances assumed, and nonparametric test was used if not. The chi-squared test or Fisher exact test was used for categorical variables. Real decline in MMSE score was calculated by measuring MMSE score minus baseline MMSE score. Expected decline of MMSE were calculated by a formula produced by previous data [14]. The analyses of the efficacy measures were based on the observed case population. For all outcomes, we used linear mixed-effects models using restricted maximum likelihood with random intercepts to estimate between-group differences, adjusted for baseline MMSE, education, donepezil and memantine use, for each measurement time point. Estimated group differences are reported with 95% CIs and *P* values. All statistical tests were two-sided, and a *P* < 0.05 was considered statistically significant.

## Results

Table 1 shows the detailed baseline characteristics of the two groups. A total of 344 AD patients were recruited, 243 cases were assigned to CT + H, and 101 cases to CT. No differences were found between the two groups in gender, age, but small differences in education (1.45 years, *P* = 0.03) and MMSE (1.48 points, *P* = 0.04). Most of the patients were initially diagnosed with mild to moderate cognitive impairment (MMSE 10–26, *N* = 314). Diabetes and hypertension were more prevalent in the group treated with CT + H.

**Table 1:** Demographic characteristics of AD patients at baseline visit

	CT + H (*N* = 243)	CT (*N* = 101)	Total (*N* = 344)
Male sex — n (%)	135 (55.55)	57 (56.43)	192 (55.81)
Age—yr., mean ± SD	69.61 ± 8.92	67.50 ± 10.65	68.99 ± 9.49
Education—yr., mean ± SD	11.05 ± 5.63*	9.60 ± 5.65	10.62 ± 5.66
MMSE score— mean ± SD	19.01 ± 6.37*	20.47 ± 5.07	19.44 ± 6.05
Mild — n	121	56	177
moderate — n	93	44	137
severe — n	29	1	30
ADL score—mean ± SD	22.98 ± 9.13	21.28 ± 6.56	22.54 ± 8.56
BMI—kg/m2, mean ± SD	22.36 ± 3.79	22.73 ± 3.04	22.48 ± 3.57
Smoke — n (%)	37 (15.22)	10 (9.90)	47 (13.66)
Diabetes — n (%)	19 (7.81)**	0 (0)	19 (5.52)
Hypertension — n (%)	62 (25.51)***	6 (5.94)	68 (19.76)
Donepezil — n (%)	202 (82.13)***	98 (97.02)	300 (87.21)
Memantine — n (%)	64 (26.33)*	14 (13.86)	78 (22.67)
Both Donepezil and Memantine	58 (23.86)*	12 (11.88)	70 (20.34)

CT + H, conventional therapy with herbal granule; CT, conventional therapy alone; MMSE, mini-mental state examination; ADL, activities of daily living; BMI, body mass index. The activities of daily living (ADL) contain 14 items, which cover 8 items of Lawton instrumental ADL scale and 6 items of Katz ADL scale (score 1–4), the range is 14 to 56, and higher scores indicate worse function. Some differences were observed in education, MMSE, diabetes history, hypertension history, donepezil use, memantine use and both donepezil and memantine

* *P* < 0.05, ** <0.01, *** <0.001

**Fig. 1:**
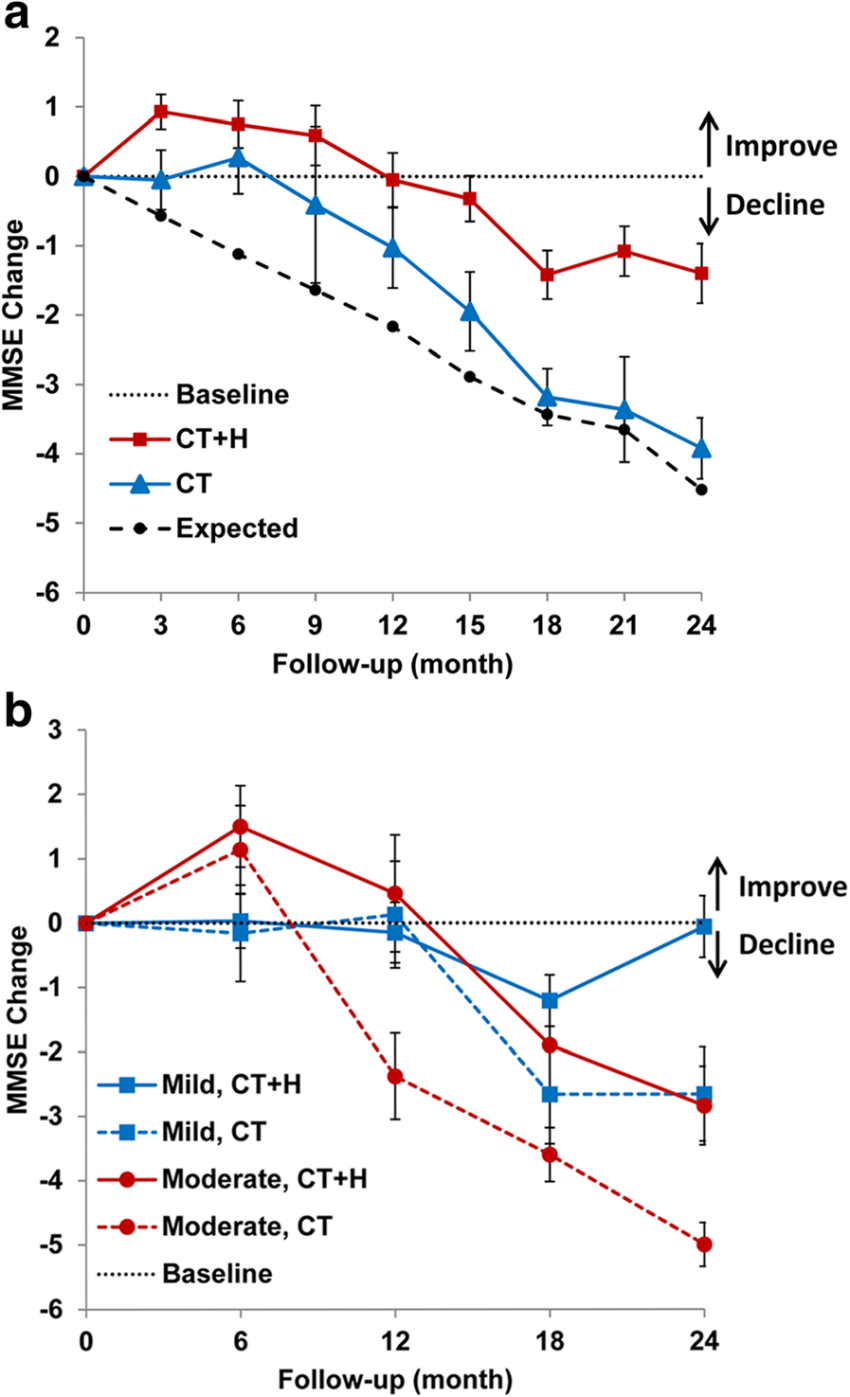
MMSE change in groups with or without herbal medicine. **a** Patients with Alzheimer’s disease had a transient improvement in cognitive function with conventional therapy (CT), but declined to a level similar to no treatment after 18 months. CT supplemented with herbal medicine (CT + H) provided additional benefit. The effect from herbal medicines became more pronounced over time. Expected decline of MMSE were calculated by formula produced from previous data. **b** In subgroup analysis, patients with moderate AD (red lines) were initially more responsive to both CT + H and CT therapies than mild AD (blue lines) patients. Over the course of treatment, CT + H outperformed CT therapy, a substantial deceleration in cognitive decline being observed in patients with moderate AD, while a long-term stabilization effect being observed in the patients with mild AD. MMSE denotes mini-mental state examination

The changes of mean MMSE scores after treatment at different visiting points were listed in Table 2. Patients treated with CT + H performed better in cognition than CT alone at different visiting points except at 9 months. Clinical improvement defined by a change of >0.0 score in MMSE lasted about 12 months by the treatment of CT + H. Moreover, the combined CT + H therapy showed a tendency of slowing the decline in cognitive function (Fig. 1a). With the treatment course prolonged, increased differences between two groups (difference in MMSE was 1.08 at 12 months, *P* = 0.009; 1.76 at 18 months, *P* = 0.010 and 2.52 at 24 months, *P* < 0.001) showed a long-term effectiveness of herbal medicine combined with conventional therapy.

**Table 2:** MMSE change between groups with or without herbal medicine

	MMSE change							
	CT + H			CT				Expected
Follow-up	N	mean ± SD	95% CI	N	mean ± SD	95% CI	P value	
3 m	121	0.93 ± 2.80	0.43 to 1.43	70	−0.05 ± 3.60	−0.91 to 0.8	0.009	−0.57
6 m	71	0.75 ± 2.90	0.06 to 1.43	33	0.27 ± 3.01	−0.79 to 1.34	0.034	−1.12
9 m	49	0.59 ± 3.01	−0.27 to 1.45	12	−0.41 ± 3.92	−2.9 to 2.07	0.077	−1.64
12 m	52	−0.05 ± 2.85	−0.85 to 0.73	28	−1.03 ± 3.12	−2.24 to 0.17	0.009	−2.17
15 m	53	−0.32 ± 2.41	−0.98 to 0.34	21	−1.95 ± 2.65	−3.16 to −0.74	0.008	−2.89
18 m	26	−1.42 ± 1.79	−2.14 to −0.69	27	−3.18 ± 2.21	−4.04 to −2.32	0.010	−3.43
21 m	23	−1.08 ± 2.36	−1.83 to −0.33	11	−3.36 ± 2.54	−5.07 to −1.65	0.014	−3.65
24 m	30	−1.40 ± 2.37	−2.28 to −0.51	26	−3.92 ± 2.26	−4.83 to −3.01	0.000	−4.52

CT + H, conventional therapy with herbal granule; CT, conventional therapy alone; MMSE, mini-mental state examination. We used linear mixed-effects models for outcome assessment, the differences were adjusted for medical history, baseline MMSE, education and use of donepezil or memantine. Follow-up was not strictly performed at all-time points; the real intervals of monitoring were different

The differences in effectiveness of AD patients with different severities were analyzed (Table 3 and Fig. 1b). Consistent with other published results, conventional therapy (CT) has a transient improvement in cognition with a peak response at 3–6 months [2, 3]. Patients with moderate AD were more responsive to CT treatment than patients with mild AD. After 6 months, however, the MMSE score of patients with moderate AD deteriorated sharply, approaching the projected trend for no treatment at 12 months (expected decline of MMSE was 3.38) [14]. Cognitive deterioration of patients with moderate AD was largely ameliorated over time when given combined CT and herbal medicine. The cognitive improvement (ΔMMSE > 0) in moderate AD with herbal medicine lasted nearly 12 months longer than CT therapy alone. In mild patients, combined therapy outperformed CT alone at almost every time point. These results indicate that the effect of herbal medicine is long-lasting. Interestingly, patients with mild AD were not initially responsive to both therapies (CT + H and CT) (<12 months). However, patients treated with CT + H exhibited a stabilization of cognitive function at 24 months (ΔMMSE = −0.06), which is a significant improvement compared to CT alone (2.60 point higher in MMSE score, *p* = 0.012). MMSE change in severe patients treated with CT + H were 1.42 ± 2.30, −1.00 ± 1.41, −0.50 ± 0.70 and −2.0 ± 0.00 at 6, 12, 18 and 24 months, respectively.

**Table 3:** MMSE change among AD patients with different severities

		MMSE change			
		6 m	12 m	18 m	24 m
Mild (MMSE 21–26)					
CT + H	mean ± SE	0.03 ± 0.42	−0.15 ± 0.47	−1.21 ± 0.40	−0.06 ± 0.48
	95% CI	−0.83 to 0.88	−1.12 to 0.81	−2.09 to −0.33	−1.1 to 0.97
	N	36	32	14	15
CT	mean ± SE	−0.16 ± 0.75	0.13 ± 0.83	−2.66 ± 0.77	−2.66 ± 0.73
	95% CI	−1.75 to 1.41	−1.66 to 1.93	−4.36 to −0.96	−4.27 to −1.05
	N	18	15	11	12
	P value	0.043	0.660	0.030	0.012
Moderate (MMSE 10–20)					
CT + H	mean ± SE	1.5 ± 0.63	0.46 ± 0.91	−1.9 ± 0.7	−2.84 ± 0.61
	95% CI	0.21 to 2.78	−1.49 to 2.42	−3.49 to −0.3	−4.19 to −1.49
	N	28	15	10	13
CT	mean ± SE	1.14 ± 0.68	−2.38 ± 0.67	−3.6 ± 0.42	−5 ± 0.34
	95% CI	−0.34 to 2.62	−3.85 to −0.91	−4.5 to −2.69	−5.75 to −4.24
	N	14	13	15	14
	P value	0.196	0.001	0.253	0.025

As severe cases were few, the analysis were done only in mild and moderate AD patients. Differences between CT + H and CT were observed at 12 to 24 months in moderate group, but later at 24 months in mild group. MMSE denotes mini-mental state examination; CT + H denotes conventional therapy with herbal medicine; CT denotes conventional therapy alone

Overall number of patients with improved (ΔMMSE ≥ 0) or deteriorated (ΔMMSE ≥ −4) MMSE scores were also analyzed (Table 4). At 24 m, 33.33% of patients in the CT + H group had improved MMSE versus 7.69% in the CT group (*P* = 0.02). On the other hand, only 16.67% of patients in the CT + H group had significant deterioration (≥4 points) in their MMSE score compared to the 65.38% of patients in the CT group (*P* < 0.001). No other adverse events were reported except for mild abdominal distension in the first few weeks in some people.

**Table 4:** Comparison of % of patients with improved or deteriorated MMSE scores

		MMSE change			
		6 m	12 m	18 m	24 m
Improved(≥0)	CT + H no.	51/71	29/52	8/26	10/30
	%	71.83	55.76	30.76	33.33
	CT, no.	23/33	10/28	2/27	2/26
	%	69.69	35.71	7.40	7.69
	P value	0.823	0.087	0.030	0.020
Deteriorated(≥4)	CT + H, no.	5/71	5/52	3/26	5/30
	%	7.04	9.61	11.53	16.67
	CT, no.	3/33	7/28	12/27	17/26
	%	9.09	25.00	44.44	65.38
	P value	0.715	0.066	0.008	0.000

CH + H denotes conventional therapy with herbal medicines; CT denotes conventional therapy alone; MMSE denotes mini-mental state examination

## Discussion

Over the past two decades, AD has been studied extensively. The causal mechanisms, however, still remain unclear. Single-target therapeutic approaches for new drug development have been challenged recently for their effectiveness and side-effects. ChEIs (such as donepezil) and NMDA antagonist i.e. memantine, the most commonly used single-target medicines, have limited long-term effectiveness for AD [2–5]. GRAPE granule as herbal mixtures with multiple targets resourced from Qifuyin in *Jing Yue Quan Shu* (First published in A.D. 1624) [15] may have a potential improvement over single-target medicines in the treatment of multifactorial, multiple cascade neurodegenerative process like AD.

So far, many complementary and alternative medicines have been assessed with promising results [7]. GAPT, a mixed Chinese herbal extracts, has shown effectively decrease the level of GSK-3β expression and Aβ accumulation via the inhibition of γ-secretase (presenilin-1) and promoting insulin degrading enzyme (IDE) and neprilysin (NEP) in APPV717I transgenic mice [16, 17]. Other possible mechanisms also have been explored [18, 19]. Based on these previous studies, GRAPE granule with analogous activity and active components to GAPT may make additional benefits to AD patients with the treatment of donepezil or memantine. Single herbal granule used in clinical settings is standardized by licensed pharmaceutical company in China (In this study, Beijing Tcmages Pharmaceutical Co. LTD., www.tcmages.com), although the composition of herbal prescription is based on doctors’ experiences [20]. The active components of the GRAPE formula fall into three correlated categories: bushen(补肾) herbs (energy-boosting with *Panax ginseng*, Rehmannia glutinosa and Epimedium brevicornu), huatan(化痰) herbs (anti-inflammation with Acorus tatarinowii and Polygala tenuifolia) and huoxue(活血) herbs (circulation-promoting with *Curcuma aromatica*, Salvia miltiorrhiza and Angelica sinensis). Those herbs above have been given to humans for thousands of years in China, and no apparent side effects have been reported.

The result of our conventional therapy without herbal medicine is consistent with other controlled trials. As shown in a study, donepezil treated patients got a short-term symptomatic improvement, but patients’ scores deteriorated significantly from baseline (−1.58 ± 0.42, *p* < 0.001 vs baseline) at 52 weeks [21]. A similar MMSE change was observed in this study, while *P* value didn’t reach a statistical significance (−1.03 ± 3.12, *P* = 0.09 vs baseline). In this study, the benefits made by conventional therapy combined with herbal granule in the first 12 months were significant albeit mild demonstrated using a mixed line model adjusted the baseline variables.

However, the addition of herbal granule made more significant long-term benefits for AD patients. During the period of prolonged treatment, differences in MMSE between CT + H and CT became larger (1.08 at 12 months, *P* = 0.009; 1.76 at 18 months, *P* = 0.01 and 2.52 at 24 months, *P* < 0.001). A possible explanation for this phenomenon is that donepezil targets at the neurotransmitter level, a downstream rather than upstream effector of pathophysiological processes of AD. Herbal medicine, on the other hand, targets broadly, including Aβ accumulation, neuroinflammation, tau hyperphosphorylation, glucose metabolism dysregulation, harmful gut microbiota, oxidative stress, and other factors that play important roles in the onset and progression of AD [16–19, 22, 23].

Except for cholinesterase inhibitors, blockers of the NMDA receptor, antioxidants or blockers of oxidative deamination (including Gingko biloba), anti-inflammatory agents etc. can be a choose for treating Alzheimer’s disease [24]. Combination therapy with cholinesterase inhibitors and memantine was demonstrated beneficial for cognition of moderate-to-severe Alzheimer’s disease, but not in mild-to-moderate AD subgroup [25]. Data from the ICTUS study accessed the effects of Gingko biloba supplementation in mild-to-moderate Alzheimer’s disease patients receiving cholinesterase inhibitors, some added cognitive benefits measured by MMSE rather than ADAS-cog was observed at 12 months but not at 6 months [26]. Like our findings, added benefits by botanical medicine are time dependent. This retrospective analysis implies that a prospective study for accessing additive effects of herbal medicine in patients with mild-to-moderate AD may have a reasonable follow-up of at least 12 months.

The analysis was done by the observed case population, selection bias is the main concern of this retrospective analysis. A prospective randomized controlled trial is needed to confirm the findings in this retrospective analysis of real world data. Another limitation of this study was set by use of MMSE as the effect indicator. Some items are judged to be easy and the ceiling effects lead to a relatively low sensitivity for detecting the cognitive change in mild AD [27]. Therefore, MMSE may be a bias factor for the result of relatively stable cognitive in mild group. The subsetting approach of analyzing ADAS-cog data suggested by other study may be an alternative in future for gaining information about treatment effects on cognitive performance in mild AD patients [28].

## Conclusions

Conventional therapy such as donepezil and memantine got a short-term symptomatic improvement, but patients’ global cognition deteriorated significantly after about 9 months of treatment compared with baseline. Combined conventional therapy with Chinese herbal medicine may bring additional longer symptomatic benefit for patients with mild-to-moderate AD.

## Abbreviations

AD: Azlheimer’s disease
ADAS-cog: Alzheimer’s disease assessment scale cognitive subscale
ADL: Abilities of daily living
BMI: Body mass index
ChEI: Cholinesterase inhibitors
CT + H: Conventional therapy combined with herbal therapy
CT: Conventional therapy
DSM-IV: Statistical manual of mental disorders
HIS: Hachinski ischaemic score
MMSE: Mini–mental state examination
MRI: Magnetic resonance imaging
NMDA: N-methyl D-aspartate
